# Simple colorimetric paper-based test strip for point-of-use quality testing of ethanol-based hand sanitizers†

**DOI:** 10.1039/d3ra08110a

**Published:** 2024-03-11

**Authors:** Aya M. El-Hassanein, Fotouh R. Mansour, Sherin F. Hammad, Aya A. Abdella

**Affiliations:** a Department of Pharmaceutical Analytical Chemistry, Faculty of Pharmacy, Tanta University Elgeish Street, the medical campus of Tanta University Tanta 31111 Egypt aya.atef.86@pharm.tanta.edu.eg Aya.atef.86@gmail.com aya150001@pharm.tanta.edu.eg fotouhrashed@pharm.tanta.edu.eg sherinhammad@pharm.tanta.edu.eg +2 0403335466 +2 0155405233

## Abstract

A novel, simple, affordable, and reliable colorimetric paper-based analytical device (PAD) was developed for the point-of-use quality testing of ethanol-based hand sanitizers, mainly against adulteration by water. The principle was based on the novel solvatochromism of methylparaben (MPB)–Fe^3+^ complex, where water is essential for complex formation and ethanol is necessary for MPB solubility. The intensity of the formed violet color, measured at 528 nm, showed a good correlation (*R*^2^ = 0.996) with the percentage water in the reaction media over a range from 40% to 100% (0–60% ethanol), with excellent accuracy and precision as indicated by the percent recovery within 100.00% ± 2% and %RSD of <2%. A PAD was prepared by the sequential immobilization of Fe^3+^ ions and MPB on chitosan-modified filter paper. The developed PAD was successfully applied for the quality testing of ethanol-based hand sanitizers using an established color index, where clearly distinct colors were observed as a function of the percentage ethanol (0–100%). The developed test strips could achieve on-site lab-quality results without expensive or sophisticated instruments using a few milligrams of FeCl_3_ and MPB in addition to regular filter paper. Accordingly, it can be used as a test strip for the quality checking of ethanol-based hand sanitizers by end users.

## Abstract 2

A novel, simple, affordable, and reliable colorimetric paper-based analytical device was developed for the point-of-use quality testing of ethanol-based hand sanitizers, mainly against adulteration by water.

## Graphical abstract

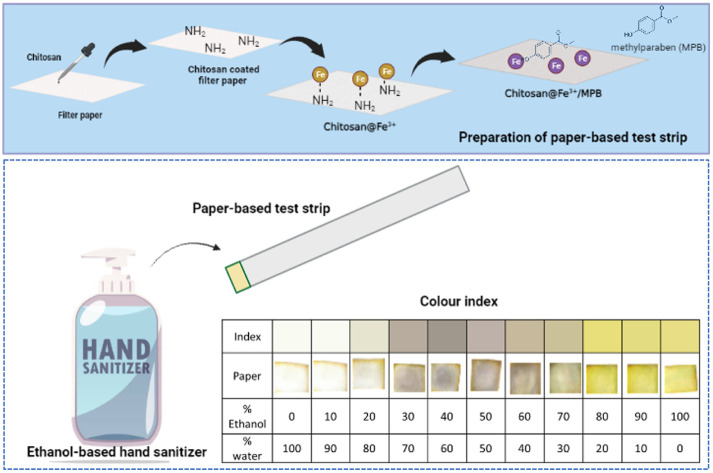

## 1. Introduction

The detection and quantification of water in organic solvents are critical in chemical processes, industrial applications, and the quality control of products.^1^ The water content in organic solvents has been determined using Karl Fischer titration,^2,3^ gas chromatography (GC),^4,5^ electrochemistry,^6–8^ and ^1^H NMR spectroscopy.^9^ However, these techniques require moisture-free conditions, expensive and sophisticated instruments, and highly qualified personnel. In addition, they tend to be tedious, expensive, and time consuming. This has motivated researchers to develop a number of dyes whose optical properties are sensitive to water,^10–13^ in addition to different fluorescent probes based on metal nanoclusters,^14^ carbon dots (CDs), and metal–organic frameworks (MOFs).^15^ Nevertheless, the generally complicated chemical synthesis and purification steps required, in addition to the limitations of dye-based sensors, including photobleaching, toxicity, and limited solubility, render them practically non-applicable.

In particular, the determination of the water content in ethanol has gained special interest in industries related to fuel, alcoholic beverages, and solvents.^16^ During the COVID-19 pandemic, the use of ethanol-based hand sanitizers (EBHSs) significantly increased worldwide among the public as well as healthcare workers with an aim to help prevent the spread of SARS-COV-2, the causative virus of COVID-19.^17^ Hand hygiene is one of the primary preventive measures to prevent the spread of such harmful germs. Although EBHSs are effective hand hygiene products, the appropriate use of such products is necessary to ensure their effectiveness.^18^ Unfortunately, at time of a critical shortage of supply, EBHSs are vulnerable to adulteration by dilution. Therefore, the Food and Drug Administration (FDA) has set a limit that the ethanol concentration should not be less than 60% (v/v) using sterile or distilled water as a diluent for it to be effective against the coronavirus.^17,19^ Therefore, the point-of-use (POU) detection of water in EBHSs could enable consumers to check product quality to ensure the maximum benefit. POU devices are commonly used by non-experts in a sample-to-answer format, in which the user loads a sample and then obtains a result.^20^ Accordingly, it is necessary to develop simple, rapid, practically applicable, and accurate procedures for the POU determination of the water content in ethanol. This would help the detection of EBHS adulteration and ensure the efficiency of EBHSs.

Colorimetric paper-based analytical devices (PADs), including dipstick test strips and lateral flow assays, have recently emerged to enable rapid, real-time, equipment-free, and inexpensive on-site lab-quality detection and even quantification of different chemical species.^21,22^ Using PADs, analyte concentration can be determined either through a reference color index (*e.g.* pH indicator strips)^23^ or smartphone-based detection.^24,25^ Numerous studies have exploited PADs to develop point-of-care and POU testing platforms.^26^ Such testing systems have been performed using immunosorbents,^27^ molecularly imprinted sorbents,^28^ or hydrophobic paper^29^ coupled with colorimetric or spectrofluorimetric detection. Nevertheless, we performed a literature survey that revealed that no PAD is available for the POU determination of the water content in ethanol.

There have been considerable investigations on the role of water in metal–ligand complexation.^30^ Water plays a well-known role in complex reactions through energy transfer and catalytic effects.^31^ In addition, water coordination in metal–ligand complex formation has been reported in numerous studies.^32^ Accordingly, some metal–ligand complexes exhibit different colors when different solvents are used, a phenomenon known as solvatochromism. This phenomenon has been extensively exploited in water determination in organic solvents employing laboratory-prepared dyes, which is tedious, environmentally hazardous, time consuming, and requires large amounts of chemicals and long synthetic procedures.^33^ Recently, a smartphone-based colorimetric sensor for the rapid determination of the water content in ethanol was developed by Shahvar *et al.*,^34^ which is based on the solvatochromism exhibited by cobalt(ii) chloride. However, this method suffered from a narrow linearity range (0.05–2.00%); therefore, it could not be extended to develop a PAD. In this context, the solvatochromism of metal complexes can provide a versatile solution, especially when using slightly soluble ligands. However, its use for water determination using a paper-based PAD has never been reported in the literature. This can be attributed to the challenging complex formation on the paper surface, which necessitates modification of the paper surface to allow chemical adsorption of both a metal and ligand. Moreover, their solubility in the tested solvents should also be considered.

In this study, we exploited the affinity of chitosan (CHT) for metal adsorption to achieve the chemical fixation of Fe^3+^ ions and subsequent complexation with methylparaben (MPB) on the paper surface. A novel, simple, and cost-effective colorimetric PAD for the full-range determination of the water content (0–100%) in ethanol was developed. The proposed sensing platform was based on the solvatochromism exhibited by the MPB–Fe^3+^ complex. The developed strategy was adapted for the construction of a PAD sensor to be used for POU quality testing of EBHS through an established color index. The developed PAD responded to water in a concentration-dependent manner. The quality testing of EBHS using the developed test strip is considered simple, affordable, rapid, and does not require a long synthetic procedure or sophisticated and expensive instruments such as GC-MS and ^1^H NMR or a Karl Fisher titrator. To the best of our knowledge, this is the first report of a PAD for POU determination of water in ethanol and quality testing of EBHS. In addition, this is the first report of solvatochromism of an MPB–Fe^3+^ complex and first time to exploit it in water content determination.

## 2. Materials and methodology

### 2.1 Chemicals and materials

MPB was supplied by Pharmalog (China). Absolute ethanol (99.8%) of HPLC grade was purchased from Sigma-Aldrich (MO, USA). Double-ring filter paper 102 (9.0 cm diameter) was used for the test strip preparation (China). Anhydrous ferric chloride was supplied by SRL Chemicals (Mumbai, India), while chitosan (CHT) (medium MW, deacetylation degree 75–85%) was purchased from Sigma-Aldrich (Missouri, USA). EBHS products, labeled to contain 70% ethanol in distilled water, were purchased from a community pharmacy. All the chemical reagents were used without further purification.

### 2.2 Instruments

UV-vis spectra were recorded in the range of 400–800 nm using a Jasco V-530 UV/vis double beam spectrophotometer (Tokyo, Japan). A DAIHAN hot plate magnetic stirrer (Batam, Indonesia) was used. All the materials were weighed using a Sartorius BP221S 4-digit analytical balance (Göttingen, Germany). A smartphone equipped with a 12-megapixel camera and the Android 13.0 operating system (Samsung Galaxy note 10 lite, Vietnam) was used for photo acquisition.

### 2.3 General procedure for determining water content using the proposed strategy

In a 5 mL volumetric flask, 0.5 mL of freshly prepared 0.5 M aqueous FeCl_3_ solution was mixed with 0.5 mL of 30 mg mL^−1^ MPB ethanolic solution. The solution was made up to the total volume using standard ethanol solutions with varying the water content from 0% to 100%. The absorbance was recorded at 528 nm and plotted against the corresponding water content (%) to construct the calibration curve.

### 2.4 Preparation of paper-based test strips and color index construction

**Scheme 1:**
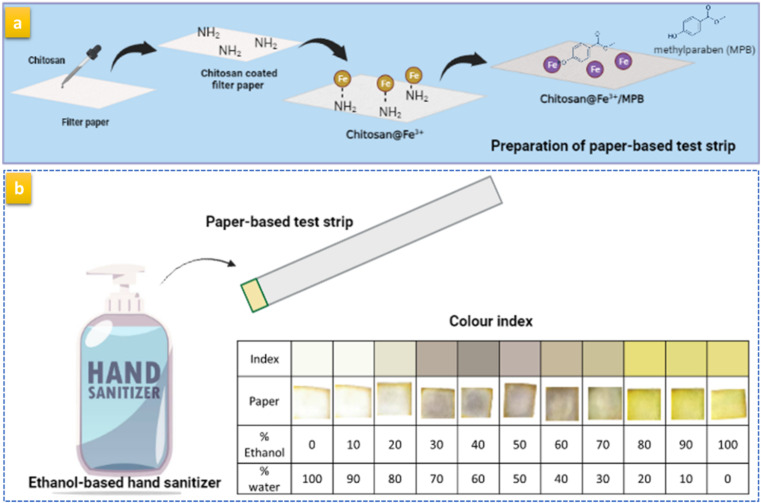
Diagrammatic representation of (a) the procedure for preparation of the paper-based test strip and (b) determination of the water content in EBHS using the prepared test strip employing the developed color index.

For the preparation of PAD, the filter paper was modified with 0.4% CHT and left to dry completely at room temperature.^35^ Then, 2 mL of 0.08 M FeCl_3_ was placed on the paper surface and allowed to dry. Finally, 1 mL of 100 mg mL^−1^ MPB was added to the paper surface and left until completely dry. The prepared paper was then cut into small square pieces (1 × 1 cm) and stored in a dry clean container until further use (Scheme 1(a)).

To develop the color index, a volume of 10 μL of ethanolic solution containing between 0% and 100% water was added to the prepared test strip. The developed colors were used to construct a color index by acquiring photos of the test strips at each percentage water content (Scheme 1(b)).

## 3. Results and discussion

**Fig. 1:**
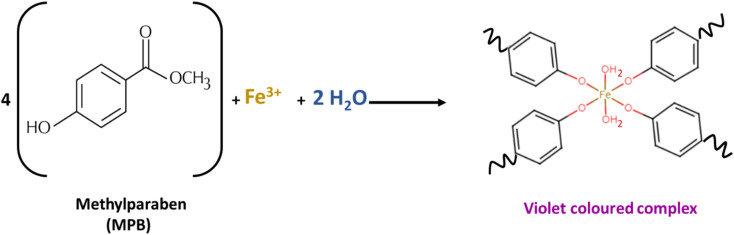
Proposed reaction equation for methylparaben reacting with FeCl_3_, showing the participation of water in the coordination complex.

**Fig. 2:**
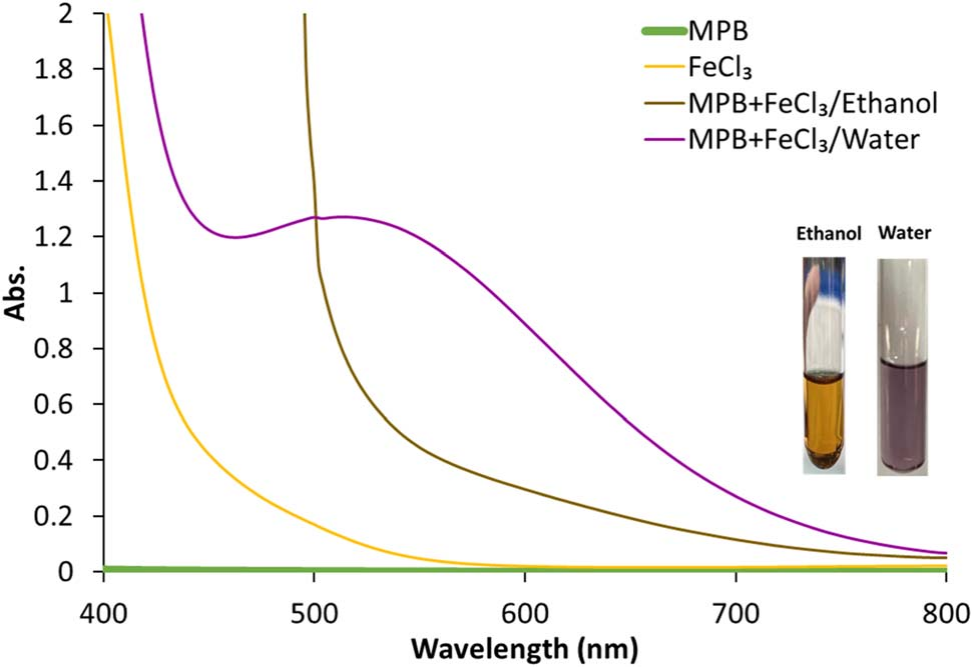
Absorption spectra of methylparaben (MPB, green), FeCl_3_ (yellow), MPB–FeCl_3_ complex in ethanol (brown) and MPB–FeCl_3_ complex in water (violet). (Inset photos): colored solutions using water (right) and ethanol (left).

MPB is a methyl ester of *p*-hydroxy benzoic acid (Fig. 1). It is sparingly soluble in water (2.5 mg mL^−1^) and freely soluble in ethanol.^36^ As a phenolic compound, MPB is expected to interact with FeCl_3_ to produce a violet-colored complex^37^(Fig. 1). However, because of its limited water solubility, MPB has never been reported to positively interact with FeCl_3_. Fig. 2 shows the visible spectrum of the MPB–Fe^3+^ complex (Fig. 2 inset: violet solution) formed in the presence of water compared with the brown solution obtained in the absence of water. Therefore, water is an important component in the formation of the MPB–Fe^3+^ complex. Thus, for this reaction to occur, a mixture of water and ethanol should be used as the solvent, where water is essential for the complex formation reaction and ethanol is necessary to maintain the solubility of MPB. Moreover, water can be involved in the coordination complex, and it is also part of the solvent that can affect the energy state of the formed complex through the dielectric constant and hydrogen bonding.^38,39^ Therefore, the molar-ratio method was applied to determine the stoichiometry of Fe^3+^ ions and MPB in the complex formation, as shown in Fig. S1.† In the molar-ratio method, the concentration of MPB was kept constant at 0.2 M while the Fe^3+^ concentration was varied between 0.01 and 0.1 M. The optimum molar concentration of Fe^3+^ ions was found to be 0.05 M, corresponding to an Fe^3+^ : MPB molar-ratio of 1 : 4. Based on the coordination number of Fe^3+^, a suggested reaction equation is proposed where two water molecules were supposed to participate in the coordination complex (Fig. 1).

The formed violet complex displayed a maximum absorbance at 528 nm at which the intensity of the absorbance was dependent on the percentage of water in the reaction medium. This solvent-dependent color formation was exploited to develop a novel solvatochromism-based sensing strategy for the determination of water content in ethanol. The developed strategy was adapted to establish a PAD for the POU determination of water. This was attempted by FeCl_3_ fixation on a CHT-coated paper strip, followed by its subsequent complexation with MPB to develop a violet color that disappears upon drying.

### 3.1 Development and optimization of the sensing strategy

**Fig. 3:**
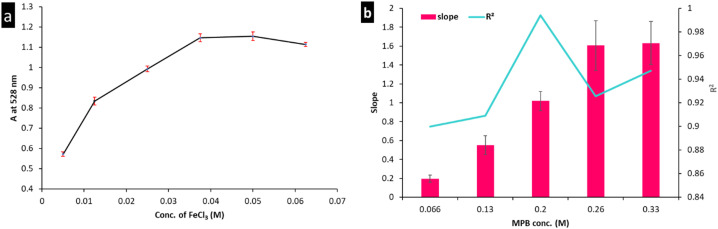
Optimization of the colorimetric reaction conditions. (a) Color intensity at 528 nm as a function of FeCl_3_ volume. (b) Maximization of the sensor sensitivity and reliability expressed by the slope and coefficient of determination (*R*^2^).

The influences of FeCl_3_ and MPB concentrations were evaluated using the univariate method to ensure that the experiment was conducted under optimal conditions. Fig. 3(a) shows the effect of varying the FeCl_3_ concentration (0.005 to 0.0625 M) on the color intensity measured at 528 nm. The maximum response was achieved using FeCl_3_ concentrations higher than 0.04 M. A volume of 500 μL was used to maintain the FeCl_3_ concentration at 0.05 M and ensure robustness (Fig. 3(a)). Moreover, MPB concentrations between 0.066 and 0.33 M were studied to determine the minimum MPB concentration that would achieve the highest sensitivity (in terms of slope) and best linearity (expressed as the coefficient of determination, *R*^2^). A calibration curve was constructed using each MPB concentration, and regression parameters were estimated. Both the slope and *R*^2^ were plotted against the corresponding MPB concentration, as shown in Fig. 3(b). According to the results presented in Fig. 3(b), an MPB concentration of 0.02 M was chosen as optimum, which met the determined reaction stoichiometry (1 : 4). At higher MPB concentrations, much higher slopes were obtained; however, the *R*^2^ values were <0.99, which would impair the strategy reliability. This could be ascribed to the decreased stability of the formed complex when the ligand concentration exceeds the optimum value. On the other hand, at MPB <0.2 M, an insufficient ligand concentration could account for the obtained *R*^2^ values.

The proposed strategy was validated according to the ICH-Q2 (R1) guidelines.^40^ In terms of linearity, the response was found to be linear over a concentration range from 40% to 100% water. A good correlation was indicated by a coefficient of determination >0.99, as shown in Fig. S2.† Regression parameters were calculated and are presented in Table 1. The accuracy of the proposed sensing strategy was evaluated using the recovery results at three different concentration levels, 40.00%, 60.00%, and 80.00%. As presented in Table 2, accuracy was indicated by the mean percentage recovery within 100.00% ± 2% at all concentration levels. Moreover, both intraday and interday precisions were indicated by a %RSD of less than 2% for all concentrations, as presented in Table 3.

**Table 1:** Regression parameters for the determination of water content in ethanol using the proposed colorimetric strategy

Parameter	Value
Linearity range (%)	40–100%
Coefficient of determination (*R*^2^)	0.996
Slope ± SD	1.07 ± 0.04
Intercept ± SD	−0.029 ± 0.02
Residual SD	0.018

**Table 2:** Accuracy of the proposed colorimetric sensor for determination of the water content in ethanol

Conc. added (%)	Conc. found (%) (*n* = 3)	% recovery	Mean percent recovery ± SD
40	39.96	99.90	99.48 ± 1.23
	40.18	100.45	
	39.24	98.10	
60	59.66	99.43	100.46 ± 1.52
	61.33	102.22	
	59.84	99.73	
80	79.16	98.95	100.02 ± 1.48
	81.37	101.71	
	79.53	99.41	

**Table 3:** Intra- and interday precision results for the determination of water content in ethanol using the proposed colorimetric sensor

Conc. added (%)	Intraday precision			Interday precision		
	Conc. found (%) ± SD (*n* = 9)	% mean recovery ± SD	%RSD	Conc. found ± SD (*n* = 9)	% Mean recovery ± SD	%RSD
40	39.79 ± 0.49	99.48 ± 1.23	1.23	39.92 ± 0.11	99.76 ± 0.56	0.57
60	60.28 ± 0.92	100.46 ± 1.52	1.52	59.94 ± 0.31	99.81 ± 0.85	0.85
80	80.02 ± 1.18	100.02 ± 1.48	1.48	79.91 ± 0.31	99.77 ± 0.27	0.27

According to these findings, the developed MPB–Fe^3+^ colorimetric sensor could successfully be used for the determination of water content in ethanol with excellent accuracy and precision using simple procedures, a small number of reagents, and short preparation and reaction times. Additionally, the developed sensing platform required neither sophisticated nor expensive instruments, as compared with the reported methods for water content determination summarized in Table S1.†

### 3.2 Development and optimization of a paper-based test strip

CHT is a polyglucosmine polysaccharide that possesses an elevated chelating capacity, mainly due to the large number of primary amino groups regularly distributed along its chain.^41^ In particular, Fe^3+^ ions interact with CHT through complexation.^42^ Therefore, the test paper was coated with CHT so that Fe^3+^ ions could be chemically adsorbed on its surface instead of requiring physical entrapment on uncoated paper to afford the PAD with good stability and reproducibility. Different CHT concentrations were tested (0.1–0.5%). The intensity of the developed color was observed at each concentration. It was noticed that the intensity of the developed color was directly proportional to the CHT concentration, reaching its maximum at 0.4%, as shown in Fig. S3(a).† At 0.5% CHT, the color intensity was markedly diminished due to the reduced permeability and wettability. Moreover, different FeCl_3_ concentrations (0.08–0.5 M) were studied. As shown in Fig. S3(b),† 0.08 M FeCl_3_ was sufficient to produce a clear violet color with a minimum yellow color in the background. Apparently, higher FeCl_3_ concentrations would obscure the appearance of a clear violet tinge. Furthermore, the concentration of the MPB loading solution was investigated (between 20–100 mg mL^−1^), showing 100 mg mL^−1^ is optimum to produce a clearer violet color (Fig. S3(c)†).

### 3.3 Application of the developed test strip for the quality testing of ethanol hand sanitizers

**Fig. 4:**
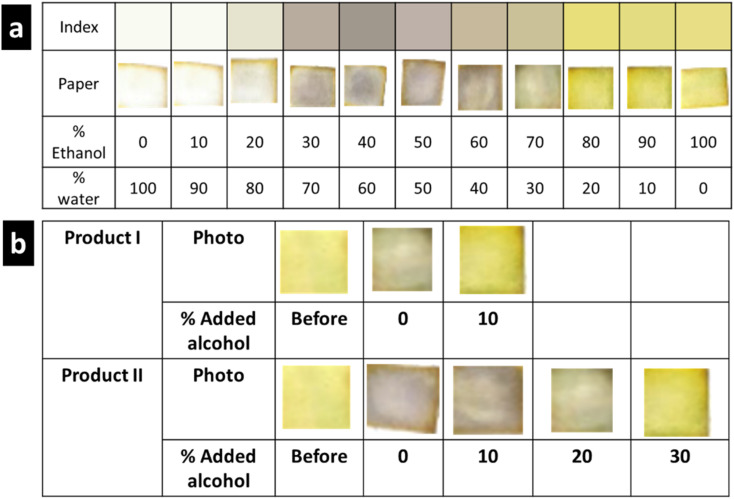
Developed paper-based test strip. (a) Established color index showing the developed color at each water percentage, (b) application of the test strip sensor for the quality testing of ethanol-based hand sanitizers.

The developed PAD prepared using the optimum conditions was used to establish an index correlating the percentage water (or %ethanol) with the developed color. Fig. 4(a) demonstrates the established color index for water contents between 0% and 100%. Notably, three clearly distinct colors were observed: golden yellow, 0–20%; white, 20–70%; and violet, 90–100% water, which corresponded to white, 0–10%; violet, 20–70%; and golden yellow, 80–100% ethanol. Moreover, the intensity of the developed violet color was dependent on the water:ethanol ratio in the standard solution. This could be ascribed to the insolubility of MPB at very high water concentrations and the instability of the formed complex at water concentrations below 30%. Because the paper was prepared by applying 10 μL of 100 mg mL^−1^ of MPB and only 10 μL of the sanitizer solution was added to the paper, the concentration of MPB (100 mg mL^−1^) exceeded its reported water solubility (2.5 mg mL^−1^). Thus, this small amount of water was not sufficient to dissolve the loaded MPB. On the other hand, starting from 30% to 70% alcohol, we found that violet color appeared and the intensity of the color increased to reach a maximum with 50% alcohol concentration. This can be explained by the presence of alcohol with a sufficient volume to dissolve the highest amount of MBP and water sufficient to allow complex formation to make the color appear strongly. At higher water percentage, *i.e.*, 60% and 70%, the intensity of the developed color starts to fade due to the decrease in the amount of water, which is essential for complex formation. According to these results (Fig. 4(a)), the appearance of a violet tinge indicated that the concentration of ethanol is ≤70%, while solutions containing a higher ethanol percentage (80–100%) produced a golden yellow color. The reproducibility of the developed test strip was investigated by comparing the resulting color in five different determinations throughout the entire range (0–100%) (Fig. S4†).

The established color index was used to determine the ethanol concentration in the EBHS products. The results are shown in Fig. 4(b), where the amount of ethanol added to the EBHS solution until an obvious color change was observed was estimated. Accordingly, the water content (%) in products I and II was found to be ≈30% and 50%, respectively. The obtained results were consistent with those obtained from spectroscopic measurements using standard addition calibration (Fig. S5(a) and (b)†).

Compared to the most popular methods for determining water content with our test strip, our developed test strip does not require specialized equipment as other methods. In contrast to carbon dots, for instance, the steps in our method are quite simple and involve no hazardous materials or extensive preparation. Moreover, it has a good detection limit and a wider linearity range than the other techniques. In addition, our method relies entirely on inspection by the naked eye, thereby avoiding the need for any instruments to detect a change in color. Therefore, it can be a good candidate for the POU determination of water content by end users.

## 4. Conclusion

A novel, simple, cost-effective, and reliable colorimetric sensing strategy was developed for the determination of water content in ethanol. The developed strategy enabled the determination of water content between 40% and 100% with acceptable accuracy and precision as indicated by a percentage recovery of 100.00% ± 2% and %RSD of <2%. Moreover, the developed strategy was adapted to construct a paper-based test strip for the first time, which allowed the point-of-use semiquantitative determination of water in ethanol through an established color index. The developed test strip consumed only a few milligrams of ferric chloride and MPB in addition to regular filter paper. Moreover, the developed PAD is facile, affordable, and can achieve on-site lab-quality results without the need for expensive or sophisticated instruments.

## Author contributions

Aya M. El-Hassanein: methodology, investigation, validation, and writing the original draft; Fotouh R. Mansour: conceptualization, supervision, review, and editing; Sherin F. Hammad: supervision, review, and editing; resources; Aya A. Abdella: conceptualization, formal analysis, methodology, investigation, validation, writing review, and editing.

## Conflicts of interest

The authors declare that they have no known competing financial interests or personal relationships that could have influenced this work.

## Supplementary Material

RA-014-D3RA08110A-s001
