# Supplementary material for: Simple colorimetric paper-based test strip for point-of-use quality testing of ethanol-based hand sanitizers
Source: RSC Adv. 2024 Mar 11;14(12):8188–94. doi: 10.1039/d3ra08110a (PMC10925907; doi:10.1039/d3ra08110a)
Supplement: RA-014-D3RA08110A-s001 [file RA-014-D3RA08110A-s001.pdf]

Electronic Supplementary Material (ESI) for RSC Advances.
This journal is © The Royal Society of Chemistry 2024

Supplementary material

Novel colorimetric paper-based test strip for point of use quality testing of ethanol-based hand sanitizer

Aya M. El-Hassanein, Fotouh R. Mansour, Sherin F. Hammad, Aya A. Abdella*

Department of Pharmaceutical Analytical Chemistry, Faculty of Pharmacy, Tanta University, 31111, Egypt.

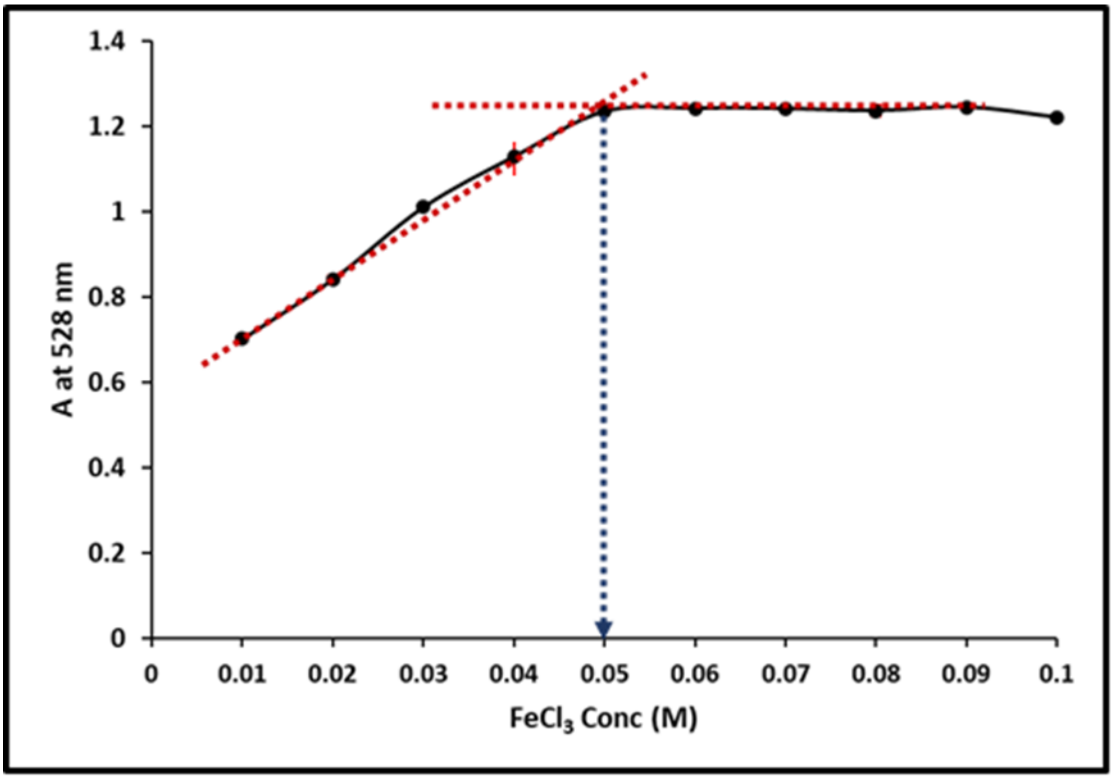

Fig. S1. Determination of reaction stoichiometry using Molar-ratio method for Fe^{3+} (0.01 - 0.1 M), and MPB 0.2 M (absorbance recorded at 528 nm).

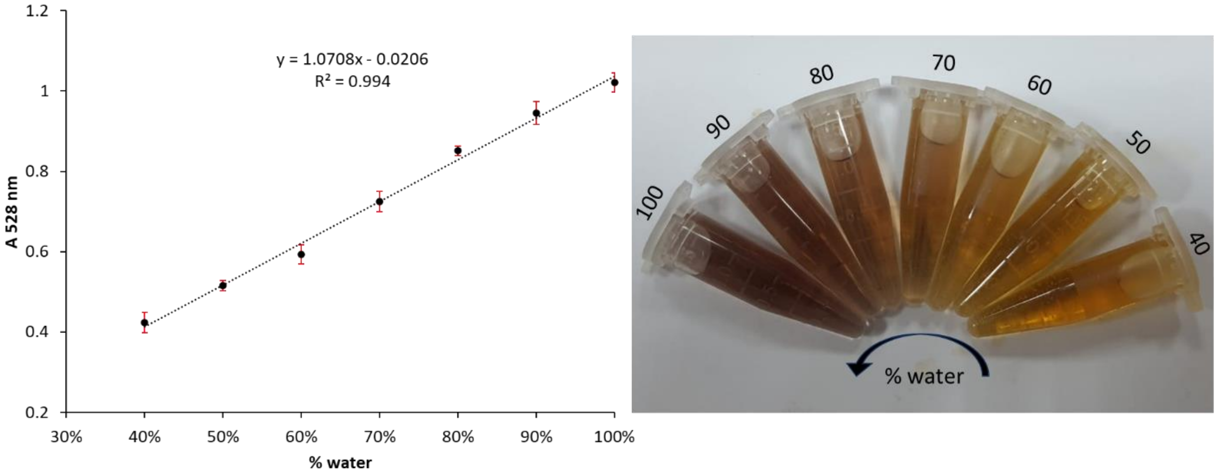

Fig. S2. The linear relationship between the developed color and water content (%) using the developed MPB-Fe^{3+} sensor within the range of 40–100% (inset: photos of color change using the developed MPB-Fe^{3+} sensor within the range of 40–100%).

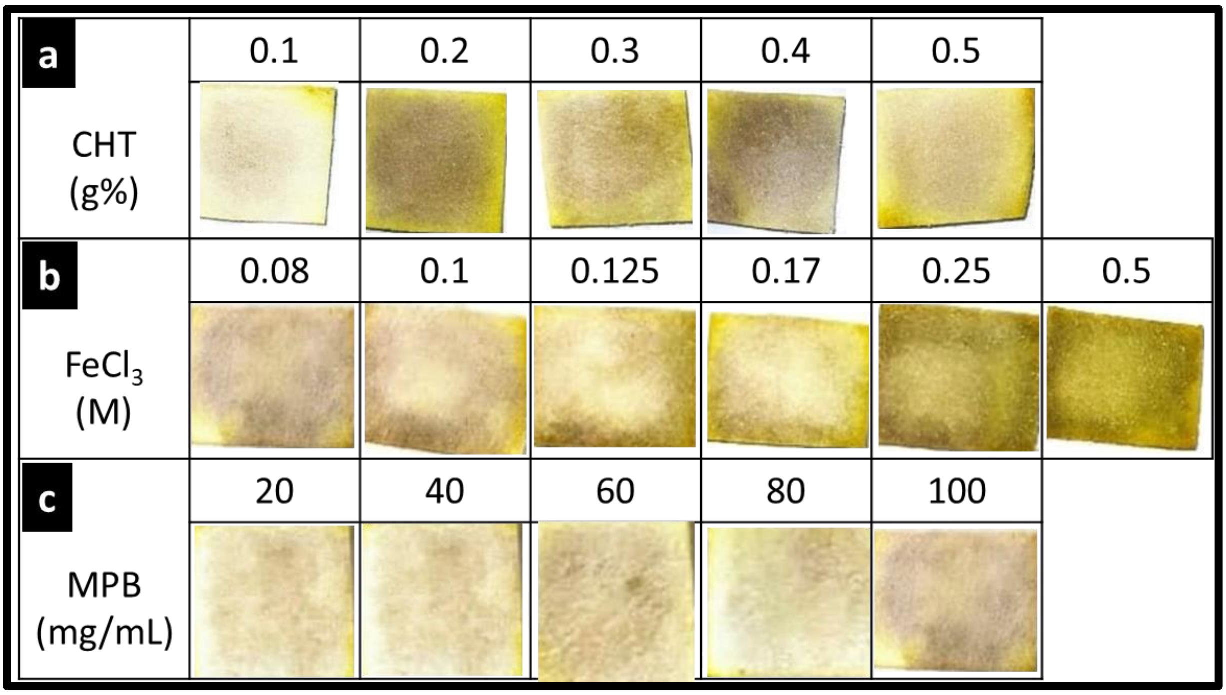

Fig. S3. Optimization of PAD preparation conditions. (a) effect of chitosan (CHT) concentration in g%, (b) effect of $FeCl_3$ concentration in moles, (c) effect of methyl paraben (MPB) concentration in mg/mL on the developed color developed using 70% ethanol.

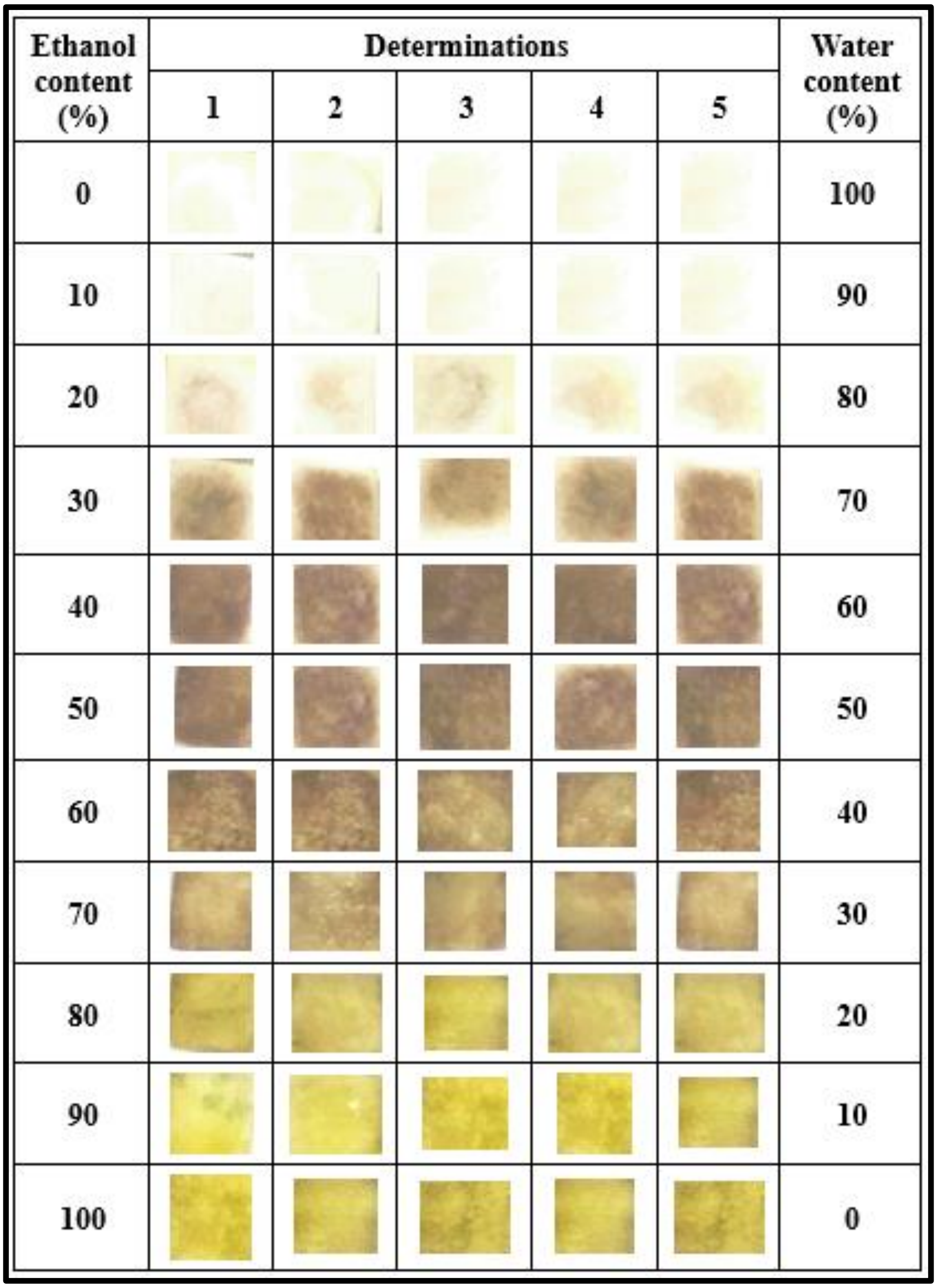

Fig. S4. Reproducibility results for the determination of water content (0-100%) in ethanol using the developed paper-based test strip.

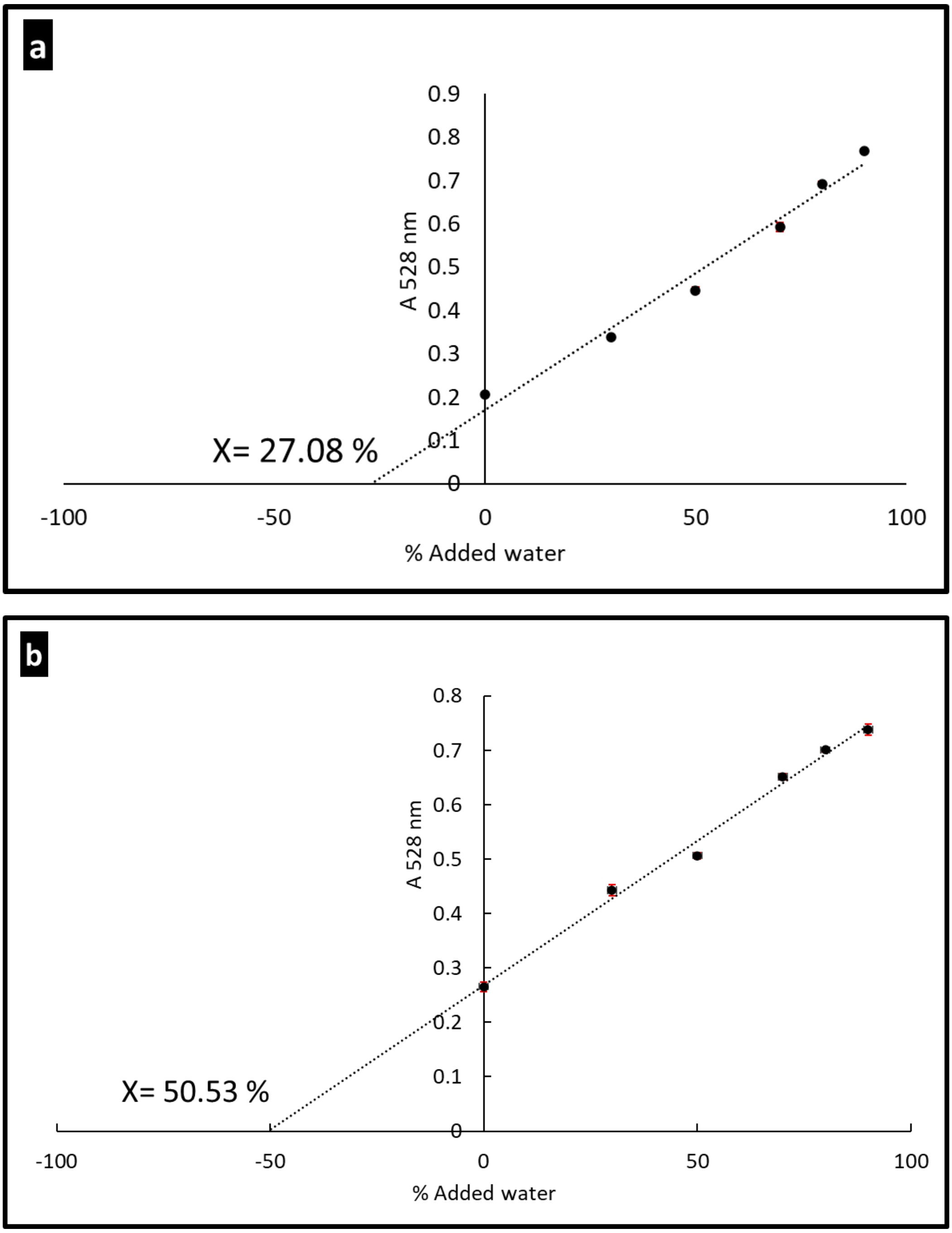

Fig. S5. Determination of water content in (a) product I and (b) product II using the proposed spectroscopic method applying standard addition calibration.

Table S1. Reported analytical methods for water content determination in different samples compared with the proposed SPCDs sensor.

Method/Principle	Sample	Time	Linearity range	DL	Ref.
**Karl Fischer titration**	Food and pharmaceutical samples	NR*	NR*	0.03%	[1]
**Spectrofluorimetric/ AgNCs**	Ethanol and DMSO	Preparation: 10 h<br>Incubation: 2 min	20-55%	4.48%	[2]
**Spectrofluorimetric/ CDs@MOF**	Organic solvents (ethanol, acetone, ACN)	Preparation: 60 min<br>Incubation: 45 min	0–70%	0.70% v/v	[3]
**^{1}H NMR chemical shifts differences**	Organic solvents	NR*	0.5-5%	NR*	[4]
**Colorimetric/ $FeCl_3$-MPB**	Ethanol and ethanol-based hand sanitizers	Preparation: 0 min<br>Incubation: 0 min	40-100%	0.088 %	This work
**$FeCl_3$/MPB@CHT-paper**	Ethanol and ethanol-based hand sanitizers	Preparation: 10 min<br>Incubation: 0 min	0-100%	-	This work

* Not reported.

References:

1 C. A. De Caro, A. Aichert and C. M. Walter, *Food Control*, 2001, **12**, 431–436.

2 N. Kong, H. Yuan, H. Zhou, Y. Zhao and S. Zhang, *Anal. Methods*, 2021, **13**, 2722–2727.

3 T. Wiwasuku, J. Suebphanpho, S. Ittisanronnachai, V. Promarak, J. Boonmak and S. Youngme, *RSC Adv.*, 2023, **13**, 18138–18144.

4 E. Kang, H. R. Park, J. Yoon, H.-Y. Yu, S.-K. Chang, B. Kim, K. Choi and S. Ahn, *Microchem. J.*, 2018, **138**, 395–400.
